# Effect of high hydrostatic pressure on the biosynthesis of sulfur amino acids in *Saccharomyces cerevisiae*

**DOI:** 10.1186/1753-6561-8-S4-P135

**Published:** 2014-10-01

**Authors:** Jimmy Soares, Fernanda Bravim, Tássia Nati, Mainã Mantovanelli Mota, James Riley Broach, Antonio Alberto Ribeiro Fernandes, Patricia Machado Bueno Fernandes

**Affiliations:** 1 Núcleo de Biotecnologia, Universidade Federal do Espírito Santo, Vitória, Espírito Santo, Brazil; 2 Penn State University College of Medicine, Hershey, Pennsylvania, USA

## 

High hydrostatic pressure (HHP) is successfully applied in several industrial segments, as in vaccine production and food conservation. The response of microorganisms to HHP treatment resemble the responses of other stresses with industrial relevance, like osmotic, temperature and ethanol, which make the HHP a valuable tool in biotechnology research, as in the ethanol production [1]. Amino acids play a key role in central metabolism besides being the building blocks of proteins, and they are important to the HHP stress response. In this study, the *Saccharomyces cerevisiae *BT0510 was exposed to 50 MPa for 30 min at room temperature, followed by incubation at room pressure with aeration for 15 min. Samples of total RNA were collected every 5 min for transcriptional analysis by DNA microarray technique. Bioinformatics analysis demonstrated the upregulation (≥ 2 fold) by HHP treatment of genes related to the sulfur amino acids synthesis, methionine and cysteine. The HHP treatment induced the genes *MET3, MET10, MET14 *and *MET16*, which are correlated with the conversion of intracellular sulfate in sulfide. *MET2*, related to the conversion of homoserine to *O*-acetylhomoserine, was also induced by HHP, as well as the gene that codes for Met17p, responsible for the incorporation of sulfide in *O*-acetylhomoserine to form homocysteine, that will be directed to methionine or cysteine synthesis. These amino acids are directly correlated with sulfur assimilation in yeast cells. Methionine is the *S*-adenosylmethionine precursor, which participates in the biosynthesis of lipids and polyamines, and is also involved in methylation reactions, being a methyl group donor [2,3]. Cysteine is part of iron-sulfur proteins and is the glutathione biosynthesis precursor. Glutathione maintain the redox state in cytoplasm, therefore, playing an important role in cell response to oxidative stress [2,3]. The key gene related to the biosynthesis of methionine (*MET6*) was upregulated by HHP, while the gene related to the biosynthesis of cysteine (*CYS4*) was unaffected. Five minutes after pressure release *MET6 *was repressed. The genes related to the conversion of methionine to *S*-adenosylmethionine, *SAM1 *and *SAM2*, were downregulated. Methionine residues are important against reactive oxygen species (ROS) [4], and genes associated with the reduction of methionine sulfoxide (*MXR1 *and *MXR2*) were induced by HHP treatment, suggesting that methionine plays an important role in the reduction of ROS resulting from stress caused by HHP [5]. Concerning the regulation of sulfur amino acids metabolism, *MET28 *was strongly induced during the entire HHP and post treatment. Other factors, such as the transcription factor encoded by *MET4 *were not affected by HHP, and also *MET30 *that negatively regulates Met4p. Met28p appears to play an important role in the biosynthesis of sulfur amino acids in response to HHP. It seems that this protein participates in the Met4 complexes-DNA stabilization. Methionine biosynthesis upregulation is not related to other stresses, such as heat and osmotic stresses, and appears to be specific to HHP, which reinforces the use of this treatment to study the stress response in microorganisms.

## References

[B1] Bravim F Lippman SI Silva LF Souza DT Fernandes AAR Masuda CA Broach JR Fernandes PMB High hydrostatic pressure activates gene expression that leads to ethanol production enhancement in a Saccharomyces cerevisiae distillery strain Appl Microbiol Biotechnol 2012 5 2093 2107 22915193 10.1007/s00253-012-4356-x PMC4771385

[B2] Ljungdahl PO Daignan-Fornier B Regulation of amino acid, nucleotide, and phosphate metabolism in Saccharomyces cerevisiae Genetics 2012 190 885 929 10.1534/genetics.111.133306 22419079 PMC3296254

[B3] Cai L Tu BP Driving the cell cycle through metabolism Annu Rev Cell Dev Biol 2012 28 59 87 10.1146/annurev-cellbio-092910-154010 22578140

[B4] Moskovitz J Berlett BS Poston JM Stadtman ER The yeast peptide-methionine sulfoxide reductase functions as an antioxidant in vivo Proc Natl Acad Sci USA 1997 94 9585 9589 10.1073/pnas.94.18.9585 9275166 PMC23225

[B5] Fernandes PMB Domitrovic T Kao CM Kurtenbach E Genomic expression pattern in Saccharomyces cerevisiae cells in response to high hydrostatic pressure FEBS Lett 2004 556 153 60 10.1016/S0014-5793(03)01396-6 14706843

